# Sanitary Science

**Published:** 1884-11

**Authors:** 


### SANITARY SCIENCE.

WHAT are proper sanitary arrangements? There is no mystery in the matter. The problem is simple enough; it is only in the application that complications arise. A great part of sanitary science can be summed up in one word, cleanliness. Clean houses, clean water, and clean air; no epidemic can resist them. This theory is as old as human knowledge. The substance of all our sanitary science accumulated by ages may be summed up in the advice of the prophet, "Wash and be clean." Not that the most profound inquiries into the origin of disease are to be undervalued. When we found that the virus of small-pox reproduced small-pox only, and that of scarlet fever bred scarlet fever only, we naturally refered their origin to a specific organism. There is a popular tradition that the body changes itself only once in seven years. The fact is, all the particles of the body change every six weeks. But the researches on the origin of disease, though vastly important, are scarcely yet within the domain of practical application. These bodies of low organized types are always associated with foulness. But whether putrid emanations are the result of the growth of these organisms, or whether the emanations form the only soil in which they can grow, no one can tell with certainty. Nor does it perhaps matter, practically, the certain thing being that if filth were prevented, none of these entozoa would remain permanently. What we mean by cleanliness is not merely personal ablution, but an uncompromising war with uncleanliness of all kinds. But to neglect of personal cleanliness epidemics might well be due. For a thousand years after the civilization of the Egyptians, the Jews, the Greeks, and the Romans faded, there was not a man or woman in Europe that ever took a bath. Hence arose the wondrous epidemics of the middle ages which cut off one-fourth of the population of Europe—the spotted plague, the black death, the sweating sickness, and the terrible mental epidemics which followed in their train—the dancing mania, the mewing mania, and the biting mania. The monks made no little mischief, imitating the foul habits of the hermits and saints of early Christian times, and the association of filth with religion led men to cease to connect disease with uncleanliness, and to resort to shrines and winking virgins for cures of maladies produced by their own physical and moral impurities.

Even now we do not understand thoroughly the processes of purification, whether natural or artificial, but we know how the atmosphere contains in itself the power of freeing itself from corrupt matter: by motion and by the attacks of oxygen upon all such matter, burning up the products of decay, and turning organic into inorganic matter. But we only half learn the lessons nature teaches us. We do not allow garbage to be thoroughly oxidized, but dig holes and store it up where the air cannot penetrate, and where the greatest facilities are offered for injurious putrefaction. Or we poison rivers with it, pouring in far more matter than can be reached by natural agents. The only safe rule is to allow none at all to be poured in. Our rivers should never be used for drainage either by manufacturers or by cities and towns. We must ventilate and we must stop overcrowding in dwellings, this last being—next to alcohol—the most prolific source of disease and crime,

**The Breath.**—Of this there are two kinds—the breath we take in, which is, or ought to be, pure air, composed, on the whole, of oxygen and nitrogen, with a minute portion of carbonic acid—and the breath we give out, which is an impure air, to which has been added, among other matters which will not support life, an excess of carbonic acid. This carbonic acid gas, when warm, is lighter than the air, and ascends; and, when at the same temperature as common air, is heavier than that air, and descends, lying along the floor, just as it lies often in the bottom of old wells or old brewers' vats, as a stratum of poison, killing occasionally the men who descend into it. Hence a word of admonition is addressed to those who think nothing of sleeping on the floor: and hence, as the poor in great cities are too apt, in times of distress, to pawn their bedsteads and keep their beds the friends of the poor—those who go about doing charitable work among them at this season—are entreated never to let this happen, and to implore them to keep the bedstead, whatever else may go, to save the sleeper from the carbonic acid stratum which lies close on the floor, especially in cold weather.

